# Absence of MERS-Coronavirus in Bactrian Camels, Southern Mongolia, November 2014

**DOI:** 10.3201/eid2107.150178

**Published:** 2015-07

**Authors:** Samuel M.S. Chan, Batchuluun Damdinjav, Ranawaka A.P.M. Perera, Daniel K.W. Chu, Bodisaikhan Khishgee, Bazarragchaa Enkhbold, Leo L.M. Poon, Malik Peiris

**Affiliations:** The University of Hong Kong, Hong Kong, China (S.M.S. Chan, R.A.P.M. Perera, D.K.W. Chu, L.L.M. Poon, M. Peiris);; Transboundary State Central Veterinary Laboratory, Ulaanbaatar, Mongolia (B. Damdinjav, B. Khishgee, B. Enkhbold)

**Keywords:** Middle East respiratory syndrome, coronavirus, MERS-CoV, bovine coronavirus, camels, dromedary, Bactrian camels, epidemiology, virology, serology, viruses, zoonoses, Mongolia

**To the Editor:** Middle East respiratory syndrome coronavirus (MERS-CoV) was first identified among humans in 2012 in Saudi Arabia (*1*). As of February 5, 2015, a total of 971 MERS cases and 356 associated deaths had been confirmed (*2*). Because MERS is a zoonotic disease, it is essential that the animal reservoirs and hosts that sustain virus circulation in nature be identified.

Seroepidemiologic and virologic studies have demonstrated evidence of MERS-CoV infection in dromedary camels (*Camelus dromedarius*) in the Arabian Peninsula (*3*), and viruses isolated from dromedaries appear capable of infecting the human respiratory tract (*4*). In some instances, MERS-CoV infection in dromedaries has preceded infection in humans (*5*), indicating that dromedaries are a natural host for MERS-CoV and a possible source of human infection. Thus, it is important to define the geographic range of MERS-CoV infection in camels and the species of camelids that are infected by MERS-CoV in nature.

Two species of camels exist: 1-hump dromedaries (*C. dromedarius*) and 2-hump Bactrian camels (*C. bactrianus*). Dromedaries are common in hot desert terrains of the Arabian Peninsula, the Middle East, Afghanistan, central Asia, India, and parts of Africa. Bactrian camels are found in colder steppes of Mongolia, Central Asia, Pakistan, and Iran. Studies have demonstrated a high seroprevalence (>90%) of MERS-CoV in adult (>5 years of age) dromedaries from the Middle East and from northern, eastern, and parts of central Africa (*6*), but whether MERS-CoV circulates among Bactrian camels is unknown. 

To determine whether MERS-CoV is circulating among both species of camels, we studied apparently healthy Bactrian camel herds in southern Mongolia during November 24–30, 2014. We investigated 11 herds in Umnugovi Province (170 sampled animals) and 1 herd in the adjacent Dundgovi Province (30 sampled animals) (Table). A convenience sample was collected from each herd; younger animals were oversampled. Serum and nasal swab samples were collected from each animal. The nasal swab samples were placed in virus transport medium and later tested by real-time PCR targeting open-reading frame 1a and upstream of envelope protein gene, as previously described (*7*); all samples were negative for MERS-CoV RNA. The serum samples were tested for the presence of MERS-CoV antibody by using a validated MERS-CoV (strain EMC) spike pseudoparticle neutralization test (*8*); no samples were positive, indicating a lack of recent or past MERS-CoV infection. A random sample of 5 serum samples each from camels in Umnugovi and Dundgovi Provinces was tested by using a microneutralization test against bovine coronavirus (BCoV) as previously described (*8*); all 10 samples were positive (titer range 1:20–1:640).

**Table:** Collection sites of nasal swab and serum specimens from Bactrian camels tested for Middle East respiratory syndrome coronavirus, southern Mongolia, November 2014

Herd no.	Province, district	Age, y			No. sampled/no. total in herd
		<1	2–4	>5	
1	Umnugovi, Khankhongor	9	5	9	23/56
2	Umnugovi, Khankhongor	7	2	9	18/31
3	Umnugovi, Khankhongor	8	0	5	13/28
4	Umnugovi, Khankhongor	0	9	17	26/65
5	Umnugov, Bayan-Ovoo	0	7	8	15/27
6	Umnugovi, Bayan-Ovoo	0	1	16	17/70
7	Umnugovi, Bayan-Ovoo	0	0	4	4/9
8	Umnugovi, Bayan-Ovoo	0	2	9	11/33
9	Umnugovi, Bayan-Ovoo	0	0	10	10/54
10	Umnugovi, Bayan-Ovoo	1	5	7	13/36
11	Umnugovi, Bayan-Ovoo	0	8	12	20/24
12	Dundgovi, Khuld	0	9	21	30/58
Total		25	48	127	200/491

The sampled animals included 127 camels >5 years of age from 12 herds across 2 provinces in southern Mongolia. Thus, the negative test results indicate that MERS-CoV is not circulating among Bactrian camels in southern Mongolia. The seroprevalence of MERS-CoV among adult dromedaries in the Middle East and Africa is typically >90%, so the lack of any serologic reactivity in camels from Mongolia implies that MERS-CoV infection does not infect Bactrian camels or that the geographic range of the virus does not extend to northeastern Asia. In contrast, infection with a BCoV-like coronavirus seems ubiquitous in Bactrian camels, as it is in dromedaries (*7*).

Dipeptidyl peptidase-4 (DPP4; cluster of differentiation 26) is the receptor for MERS-CoV. As deduced from the human DPP4–MERS-CoV spike protein structural model, the differences in the amino acids in DPP4 molecules of dromedary and Bactrian camel were found in 2 small regions far from the binding interface of DDP4 and MERS spike protein (*9*). The 15 aa of DPP4 critical for binding with MERS-CoV spike protein are conserved between dromedaries and Bactrian camels. Definitive evidence of susceptibility, or lack thereof, of Bactrian camels to MERS-CoV can be established only by experimental infection of these animals.

Even if Bactrian camels are susceptible to MERS-CoV infection, geographic separation may be an alternative explanation for the absence of MERS-CoV among camels in Mongolia. So far, Australia is the only country where dromedaries appear to be free of MERS-CoV; however, as with dromedaries elsewhere, dromedaries in Australia are infected by a BCoV-like virus (*8*). Dromedaries in Australia originated from Afghanistan; these camels were shipped to Australia in the early part of the twentieth century to work on railroad construction projects. There are 2 plausible explanations for the lack of MERS-CoV in Australia: the small numbers of adult animals that were transported from Afghanistan to Australia might not have been sufficient to introduce the virus into Australia or the virus might have been absent from dromedaries in Afghanistan.

Our study was limited by sample size and by the breadth of the study area. Mongolia has 21 provinces and ≈349,300 Bactrian camels, but we studied just 2 southern provinces and a total of 200 camels. Umnogovi Province has the largest, and Dundgovi Province the fifth largest, camel population in the country (≈113,000 and ≈28,000 animals, respectively). Further studies on the epidemiology of MERS-CoV infection in dromedaries and Bactrian camels from central Asia, China, and Mongolia are warranted.
